# Use of Health Care Chatbots Among Young People in China During the Omicron Wave of COVID-19: Evaluation of the User Experience of and Satisfaction With the Technology

**DOI:** 10.2196/36831

**Published:** 2022-06-09

**Authors:** Yi Shan, Meng Ji, Wenxiu Xie, Xiaomin Zhang, Xiaobo Qian, Rongying Li, Tianyong Hao

**Affiliations:** 1 School of Foreign Studies Nantong University Nantong China; 2 School of Languages and Cultures University of Sydney Sydney Australia; 3 Department of Computer Science City University of Hong Kong Hong Kong China; 4 Department of Linguistics Macquarie University Sydney Australia; 5 School of Computer Science South China Normal University Guangzhou China; 6 School of Artificial Intelligence South China Normal University Guangzhou China

**Keywords:** health care chatbots, COVID-19, user experience, user satisfaction, theory of consumption values, chatbots, adolescent, youth, digital health, health care, omicron wave, omicron, health care system, conversational agent

## Abstract

**Background:**

Long before the outbreak of COVID-19, chatbots had been playing an increasingly crucial role and gaining growing popularity in health care. In the current omicron waves of this pandemic when the most resilient health care systems at the time are increasingly being overburdened, these conversational agents (CA) are being resorted to as preferred alternatives for health care information. For many people, especially adolescents and the middle-aged, mobile phones are the most favored source of information. As a result of this, it is more important than ever to investigate the user experience of and satisfaction with chatbots on mobile phones.

**Objective:**

The objective of this study was twofold: (1) Informed by Deneche and Warren’s evaluation framework, Zhu et al’s measures of variables, and the theory of consumption values (TCV), we designed a new assessment model for evaluating the user experience of and satisfaction with chatbots on mobile phones, and (2) we aimed to validate the newly developed model and use it to gain an understanding of the user experience of and satisfaction with popular health care chatbots that are available for use by young people aged 17-35 years in southeast China in self-diagnosis and for acquiring information about COVID-19 and virus variants that are currently spreading.

**Methods:**

First, to assess user experience and satisfaction, we established an assessment model based on relevant literature and TCV. Second, the chatbots were prescreened and selected for investigation. Subsequently, 413 informants were recruited from Nantong University, China. This was followed by a questionnaire survey soliciting the participants’ experience of and satisfaction with the selected health care chatbots via wenjuanxing, an online questionnaire survey platform. Finally, quantitative and qualitative analyses were conducted to find the informants’ perception.

**Results:**

The data collected were highly reliable (Cronbach *α*=.986) and valid: communalities=0.632-0.823, Kaiser-Meyer-Olkin (KMO)=0.980, and percentage of cumulative variance (rotated)=75.257% (*P*<.001). The findings of this study suggest a considerable positive impact of functional, epistemic, emotional, social, and conditional values on the participants’ overall user experience and satisfaction and a positive correlation between these values and user experience and satisfaction (Pearson correlation *P*<.001). The functional values (mean 1.762, SD 0.630) and epistemic values (mean 1.834, SD 0.654) of the selected chatbots were relatively more important contributors to the students’ positive experience and overall satisfaction than the emotional values (mean 1.993, SD 0.683), conditional values (mean 1.995, SD 0.718), and social values (mean 1.998, SD 0.696). All the participants (n=413, 100%) had a positive experience and were thus satisfied with the selected health care chatbots. The 5 grade categories of participants showed different degrees of user experience and satisfaction: Seniors (mean 1.853, SD 0.108) were the most receptive to health care chatbots for COVID-19 self-diagnosis and information, and second-year graduate candidates (mean 2.069, SD 0.133) were the least receptive; freshmen (mean 1.883, SD 0.114) and juniors (mean 1.925, SD 0.087) felt slightly more positive than sophomores (mean 1.989, SD 0.092) and first-year graduate candidates (mean 1.992, SD 0.116) when engaged in conversations with the chatbots. In addition, female informants (mean 1.931, SD 0.098) showed a relatively more receptive attitude toward the selected chatbots than male respondents (mean 1.999, SD 0.051).

**Conclusions:**

This study investigated the use of health care chatbots among young people (aged 17-35 years) in China, focusing on their user experience and satisfaction examined through an assessment framework. The findings show that the 5 domains in the new assessment model all have a positive impact on the participants’ user experience and satisfaction. In this paper, we examined the usability of health care chatbots as well as actual chatbots used for other purposes, enriching the literature on the subject. This study also provides practical implication for designers and developers as well as for governments of all countries, especially in the critical period of the omicron waves of COVID-19 and other future public health crises.

## Introduction

### Background

Regretfully, more than 95% of the population suffers from particular health problems [1], and about 60% of them visit a doctor when merely affected by minor illnesses, including a cold, headache, and stomachache. Actually, 80% of these diseases can be cured with home remedies, without the intervention of a doctor [2]. In this scenario, health care chatbots are capable of monitoring people’s health [1] by providing timely, useful health care information, especially during the omicron waves of COVID-19. These conversational agents (CA) play a crucial role in health care in the fast-pacing world, where the public prefers to be addicted to social media rather than to be concerned about their health [3] and mobile phones are becoming the primary source of information. Meanwhile, chatbots are substantially alleviating the pressure on the already overloaded health care systems in various countries. Therefore, an upsurge in the development and application of health care chatbots has been witnessed since the advent of ELIZA in 1966, which served as a psychotherapist promoting communication with patients [4]. It inspired the design and application of other health care chatbots [5], including Casper [2], MedChat [2], PARRY [6], Watson Health [7], Endurance [7], OneRemission [8], Youper [9], Florence [10], Your.Md [11], AdaHealth [12], Sensely [13], and Buoy Health [14]. These leading chatbots offer patients tailored health and therapy information, recommended products and services, and personalized diagnoses and treatments based on confirmed symptoms [15]. Facing the repeated daunting waves of COVID-19, many people are craving information to respond to the coronavirus [16], which is incessantly mutating. This sudden surge in the demand for information is increasingly overtaxing health care resources [17], including various health care hotlines and clinic services, so health care chatbots seem to be the only possible solution [17,18]. Given the status quo, the user experience of and satisfaction with chatbots are more important now than ever before. Relevant studies have been undertaken in some countries to investigate the effectiveness [19], usability [20], and acceptability [21]. Depending on technology acceptance theories (TAT), these studies on the use of health care chatbots focused on improving user experience and satisfaction through personalization [22], enjoyment [19], and novelty [23]. However, almost no investigation has been conducted in this respect among people in China from the perspective of the theory of consumption values (TCV).

Chatbots display unmatched advantages compared to other health care alternatives: alleviating the pressure on contact centers [24] and reducing contact-induced risks, satisfying unprecedented needs for health care information in the case of the shortage of qualified human agents [25], providing cost-effective 24/7 service [25], offering consistent service quality [26], and making no moral judgement on undesirable information provided by users [27]. The enhancement of these qualities motivates their increased use for health care purposes. This trend is being accelerated in the repeated outbreak waves of COVID-19, where chatbots are being used to screen potential infected cases [28], to help call centers to triage patients [29], and to recommend the most appropriate solutions to patients [29].

These selling points will facilitate popularizing health care chatbots only when the public is willing to utilize them and adopt their recommendations [30,31] in the face of the rampant COVID-19 pandemic. To promote adoption and adherence, many related studies have been undertaken in terms of the use of chatbots during this global health emergency to explore user reaction [32], to probe user experience and design considerations [33], to focus on the usage purposes [34], to identify differences in chatbot feature use by gender, race, and age [35], to improve the bot response accuracy [36], to investigate people’s behavior when seeking COVID-19 information [37], and to introduce newly developed COVID-19–specific chatbots [38,39]. Apparently, few investigations [32,33] have examined the users’ perception of these chatbots, but extant studies predominantly focus on technology acceptance [40,41], neglecting user experience and user satisfaction. Admittedly, user experience and user satisfaction are crucially significant because good user experience is the prerequisite of user adoption of information systems (IS) [42,43] and user satisfaction is a crucial factor for IS acceptance intention [44,45].

To fight against the COVID-19 pandemic, chatbots have been used to provide psychological service for medical professionals and the general public in China [46]. Unfortunately, only 1 study, based on Deneche and Warren [47], investigated the user experience of and satisfaction with chatbots addressing COVID-19–related mental health in Wuhan and Chongqing, China [48]. However, this study focused on the determinants influencing user experience and satisfaction rather than on user experience and satisfaction per se. This gap in the literature needs to be filled.

### Objective

The objective of this study was twofold: (1) Informed by Deneche and Warren’s [47] evaluation framework, Zhu et al’s [48] measures of variables, and the TCV [49], we designed a new assessment model for the user experience of and satisfaction with chatbots on mobile phones, and (2) we aimed to validate the newly developed model and use it to investigate the user experience of and satisfaction with the popular Chinese and English language chatbots for timely self-diagnosis and general information concerning COVID-19 and the latest virus variants among young people (aged 17-35 years) in China in order to provide evidence for the potential improvements and developments of chatbots to sustain adherence and adoption, which is undoubtedly an inevitable worldwide trend.

Based on the twofold research aim, we proposed the following hypotheses:

- Hypothesis 1: Explaining user behaviors in terms of diverse value-oriented factors (function, emotion, social influence, and environment), the newly developed comprehensive assessment model will have a high degree of reliability and validity and can better evaluate the user experience of and satisfaction with chatbots on mobile phones.
- Hypothesis 2: The informants will generally be satisfied with their experience of using popular health care chatbots.

Two facts justify the necessity of this research: Young people (aged 17-35 years), occupying a large portion of the population in China, are more addicted to mobile health care apps than other age groups, and sustainable user adoption of and adherence to chatbots in this population can considerably emancipate clinicians, enabling them to pay close attention to more complex tasks and enhance the availability of qualified health care services to the general public in China.

## Methods

### Overall Procedures

We followed 5 steps to reveal the user experience of and satisfaction with chatbots in young people (aged 17-35 years) in China. First, we established an assessment model evaluating user experience and satisfaction based on the related literature and TCV and designed a questionnaire according to the assessment model. Second, we screened and selected the chatbots to be investigated. Third, we recruited 413 students from Nantong University, China, as informants of this study. Fourth, we collected the informants’ demographic information, tested their health literacy, and solicited their experience of and satisfaction with the selected health care chatbots via a questionnaire survey. Finally, we conducted quantitative and qualitative analyses based on the data collected through the questionnaire.

### Recruitment of Informants

Participants were recruited from among students of Nantong University, China. This university recruits around 8000 students annually, with the total number of students exceeding 30,000. On-campus psychological tests and students’ counselors reported that a large percentage of students suffer from psychological problems of varying degrees during the repeat COVID-19 outbreaks. They urgently need intelligence-based CA for self-diagnosis and general information on the pandemic and the latest virus variants to ease their psychologically strained mind during the public health emergency. Their experience of and satisfaction with health care chatbots are, on the whole, representative and characteristic of the adolescent and middle-aged population in China. The questionnaire survey was approved and supported by the school authority in charge of students’ affairs and the student participants themselves. It was conducted using the online questionnaire survey platform wenjuanxing [50] on January 8, 2022, and the survey lasted until no additional questionnaire was submitted online for 2 consecutive days (January 12, 2022). Over this period, the survey was announced to the entire student body of over 1000 at the School of Foreign Studies, Nantong University, through emails and WeChat groups. The reason informants were recruited from among these students is that only these English majors reach the English proficiency enabling them to experience the use of English language chatbots. Characteristic of all the schools of foreign studies of all colleges and universities in China, the overwhelming majority of students are female.

### Selection of Health Care Chatbots

**Figure 1:**
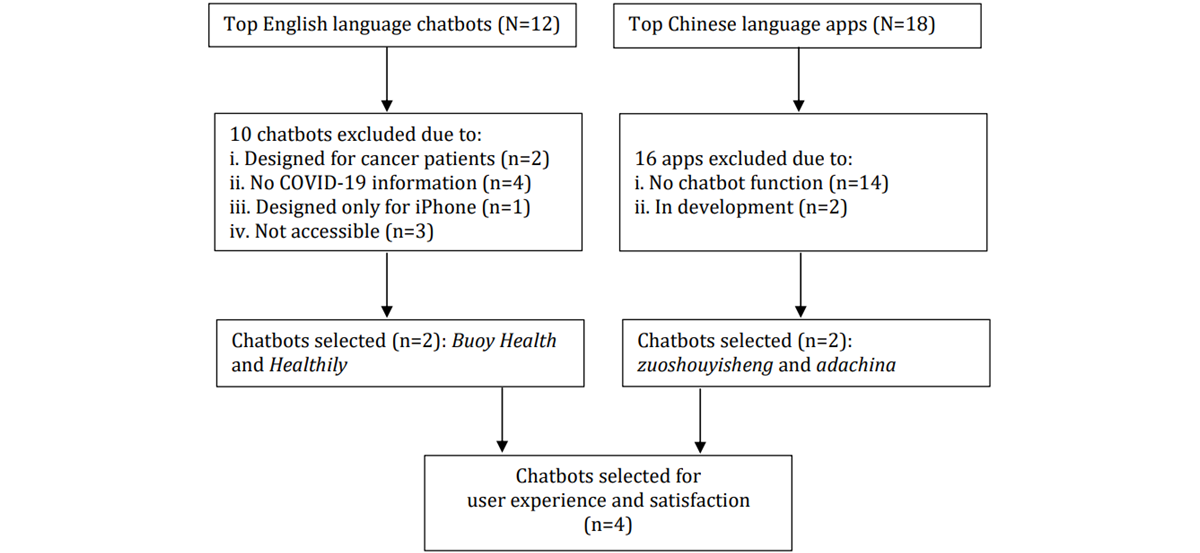
Flowchart of selecting Chinese and English language health care chatbots. Of the top 12 English chatbots, 3 (25%) were not accessible due to technical errors, requirement of enterprise/school identification, or difficult application for a demo.

First, we chose the top 12 health chatbots popular throughout the world as the scope of selection of English language chatbots. These chatbots were reviewed by name, description, function, and experience, and only 2 (16.7%) of them, *Buoy Health* [14] and *Healthily* [11], were finally chosen (Figure 1).

Subsequently, we selected leading Chinese language chatbots from the dominant Android app markets, including *360 Mobile Assistant*, *Baidu Mobile Assistant*, and *Tencent MyApp*, and the iOS App Store. The keywords *health care chatbot* (*医疗保健聊天机器人*), *health care bot* (*医疗保健机器人*), *health care app* (*医疗保健应用软件*), *health care applet* (*医疗保健小程序*), *psychological health chatbot* (*心理健康聊天机器人*), *psychological health bot* (*心理健康机器人*), *psychological health app* (*心理健康应用软件*), and *psychological health applet* (*心理健康小程序*) were searched in Chinese on January 8, 2022. The selection followed 2 steps: (1) A total of 18 apps were identified by the search words, and (2) a further review revealed that only 4 (22.2%) of these 18 apps—*zuoshouyisheng* (*左手医生*), *adachina* (*爱达健康*), *zhinengyuwenzhen IPC* (*智能预问诊IPC*), and *xiaojiuzhinengwenzhenjiqiren* (*小九智能问诊机器人*)—have the chatbot function, while 2 (11.1%; *zhinengyuwenzhen IPC* and *xiaojiuzhinengwenzhenjiqiren*) are still in development and provide no demos for experience and merely 2 (*zuoshouyisheng* and *adachina*) can truly function as chatbots. The selection process is illustrated in Figure 1.

Before answering the questionnaire, it was arranged that the informants would experience the use of both Chinese and English language chatbots for around 2 weeks. This 2-week experience was intended to guarantee the validity and reliability of the questionnaire survey.

### Assessment Model and Questionnaire

Informed by Deneche and Warren’s [47] evaluation framework, Zhu et al’s [48] measures of variables, and TCV [49], the assessment model designed for this study included 5 evaluation dimensions (functional, emotional, epistemic, social, and conditional) consisting of 18 variables (Table 1). These variables are supposed to contribute to user experience and user satisfaction. The questionnaire included 36 measures (Multimedia Appendix 1). Measures 1-26 were designed in light of the variables listed in Table 1. To solicit sufficient information, some variables may have corresponded to more than 1 measure. For example, “performance” was related to 6 measures (15-20) in the questionnaire. Measures 27-36 were intended to display the informants’ overall experience and satisfaction.

**Table 1:** Assessment model of user experience and user satisfaction.

Dimension	Variables
Functional	Context awareness Language suitability Customized service User-friendliness Performance
Emotional	Enjoyment Relief from mental disorders
Epistemic	Novelty Desire for knowledge Knowledge enrichment
Social	Engagement Empathy Human likeness Privacy
Conditional	Time Place Technological context Mental state

### Data Collection

The survey was conducted through wenjuanxing [50], an online questionnaire platform that is most popular in China. Three categories of data were collected via the online questionnaire: demographic information about the informants, their health literacy, and their experience of and satisfaction with the selected Chinese and English language chatbots. The demographic section collected data on the informants’ age, gender, grade, English proficiency, and way to obtain health care information during the COVID-19 pandemic. The health literacy part tested the informants’ basic medical vocabulary. The user experience and satisfaction module elicited data concerning the respondents’ ratings of the 36 measures. The score of each measure was rated between 1 and 4 points (1: totally agree; 2: basically agree; 3: basically disagree; 4: totally disagree).

### Data Analysis

Quantitative analyses were performed using SPSS Statistics version 22.0 (IBM Corp) and R version 4.0.2 (The R Foundation). First, the demographic data and health literacy of the participants were briefly described as the background information of the analysis. Afterward, the reliability and validity of the data concerning user experience and satisfaction were confirmed. Finally, the minimum, maximum, and mean scores, as well as SD, were calculated for each of the 36 measures, and the percentages of informants falling into each of the 4 ratings of the 36 measures were computed. Inspection of the data and residual plots for mean scores of the 36 measures did not indicate any violation of assumptions of normality, independence, and homogeneity of variance, so the correlation between measures 1-26 and measures 27-36 was tested and confirmed.

### Ethical Considerations

Nantong University approved this study. It is an official practice in this university to ask the Students’ Affairs Department for approval before collecting data from students. We followed this practice. In addition, there is no ethics review board in Nantong University. Therefore, a review number or code for this study could not be provided.

## Results

### Informant Demographics

A total of 413 questionnaires were collected, including 358 (86.68%) from female respondents. This can be explained by the fact that over 80% of students studying in the School of Foreign Studies, Nantong University, are female. The age of the participants ranged from 17 to 33 years (mean 20.96, SD 2.18). The overwhelming majority (n=402, 96.86%) of them are aged between 18 and 25 years. The informants included freshmen (n=66, 15.98%), sophomores (n=72, 17.43%), juniors (n=110, 26.63%), seniors (n=68, 16.46%), first-year graduate candidates (n=52, 12.59%), and second-year graduate candidates (n=45, 10.90%). They study in the School of Foreign Studies. Most of them (n=259, 62.71%) scored more than 100 in English in the entrance examinations for colleges and universities. Most of them (n=267, 65.65%) passed College English Test Band 6 (CET 6), Test for English Majors Band 4 (TEM 4), and TEM 8. Their English proficiency can well enable them to experience the use of English language chatbots. The majority of the informants (n=355, 85.96%) obtained COVID-19–related health care information through visiting a doctor or logging on to the internet. Table 2 shows the informants’ demographics, including grade, age, gender, and English proficiency, as well as the health care information sources they drew on.

**Table 2:** Informant demographics (N=413).

Categories			Participants, n (%)		Cumulative percentage (%)
**I’m a____.**					
	Freshman	66 (15.98)		15.98	
	Sophomore	72 (17.43)		33.41	
	Junior	110 (26.63)		60.05	
	Senior	68 (16.46)		76.51	
	First-year graduate candidates	52 (12.59)		89.10	
	Second-year graduate candidates	45 (10.90)		100.00	
**I’m ____ years old.**					
	17	2 (0.48)		0.48	
	18	27 (6.54)		7.02	
	19	56 (13.56)		20.58	
	20	89 (21.55)		42.13	
	21	79 (19.13)		61.26	
	22	65 (15.74)		77.00	
	23	47 (11.38)		88.38	
	24	19 (4.60)		92.98	
	25	18 (4.36)		97.34	
	26	3 (0.73)		98.06	
	27	2 (0.48)		98.55	
	29	1 (0.24)		98.79	
	32	3 (0.73)		99.52	
	33	2 (0.48)		100.00	
**I’m ____.**					
	Male	55 (13.32)		13.32	
	Female	358 (86.68)		100.00	
**I scored ____ in English in the entrance examinations for colleges and universities.**					
	>90	154 (37.29)		37.29	
	>100	79 (19.13)		56.42	
	>110	41 (9.93)		66.34	
	>120	75 (18.16)		84.50	
	>130	57 (13.80)		98.31	
	>140	7 (1.69)		100.00	
**I passed ____.**					
	CET^a^ 3	52 (12.59)		12.59	
	CET 4	94 (22.76)		35.35	
	CET 6	47 (11.38)		46.73	
	TEM^b^ 4	150 (36.32)		83.05	
	TEM 8	70 (16.95)		100.00	
**Facing COVID-19, I mainly obtain health care information through ____.**					
	Visiting a doctor	94 (22.76)		22.76	
	Logging on to the internet	261 (63.20)		85.96	
	Reading books, papers, and journals	14 (3.39)		89.35	
	Families, friends, and classmates	31 (7.51)		96.85	
	Health care hotlines	4 (0.97)		97.82	
	Health care chatbots	9 (2.18)		100.00	

^a^CET: College English Test.

^b^TEM: Test for English Majors.

### Data Reliability and Validity

As shown in Table S1 in Multimedia Appendix 2, Cronbach *α* (.986) for all the items (measures), except item (measure) 4, rated by all the 413 respondents was well above .9. If item 4 was deleted, Cronbach *α* increased merely by .001, so it was retained for the analysis. This indicates that the data collected for each measure in the questionnaire are highly reliable. The *corrected item-total correlation* for each measure was well above 0.4, which implies that the 36 measures are closely correlated.

The data were highly valid (Table S2 in Multimedia Appendix 2). The *communalities* for all the 36 items ranged from 0.632 to 0.823, well above 0.4, indicating that all these items are reasonable and should be included in the analysis. The value of Kaiser-Meyer-Olkin (KMO) value of 0.980 was substantially above 0.6, showing that all the data concerning the 36 items are suitable for extraction. The *percentage of variance (rotated)* for factors 1-3 was 30.428%, 28.077%, and 16.752%, respectively, and the *percentage of cumulative variance (rotated)* for the 3 factors was 75.257%, considerably above 50%. This means that all the data on all the items can be extracted validly.

### User Experience and Satisfaction

Table 3 displays the results of the descriptive analysis of user experience and satisfaction. The minimum, maximum, and mean scores were based on the rating scale of each measure (1: totally agree; 2: basically agree; 3: basically disagree; and 4: totally disagree). The mean scores of the 36 measures were lower than or slightly over 2, indicating that the respondents were inclined to totally or basically agree with these measures. In other words, they found the chatbots pleasurable and satisfactory in terms of the functional, emotional, epistemic, social, and conditional domains.

The functional domain displayed the lowest mean score (1.762, SD 0.630), closely followed by the epistemic domain (mean 1.834, SD 0.654). This indicates that the respondents were overall satisfied with the function of the selected chatbots when seeking self-diagnosis and general knowledge about COVID-19 and the latest virus variants and that they had enriched their COVID-19–related knowledge through the novel way of communication with the chatbots. The conditional, social, and emotional domains showed a similar mean score of slightly lower than 2. It follows that the participants found it necessary and technologically possible to obtain health care information through communicating with the chatbots via a mobile phone anytime and anyplace in the face of the rampant COVID-19 pandemic, which imposes on them mental stress in varying degrees. Additionally, they believed that seeking COVID-19–related health care information through communicating with the chosen chatbots was generally enjoyable and mentally relaxing and that the somehow humanlike empathetic chatbots made them socially and emotionally engaged in machine-human conversations. Furthermore, they basically thought that their personal information revealed in communication with the chatbots would be used for medical or research purposes rather than for unreasonable or even illegal ends. Overall, they had a pleasant and satisfactory experience when communicating with the chatbots for COVID-19–related self-diagnosis and health care information, as shown by the mean scores of *experience* (1.978, SD 0.639) and *satisfaction* (1.894, SD 0.617) in Table 3.

Table S3 in Multimedia Appendix 2 shows the proportion of informants falling into each of the 4 ratings of the 36 measures. Over 80% (n=330) of the respondents totally and basically agreed with all measures, except measures 3, 4, 17, 23, and 28. Strikingly, more than 90% of the respondents totally and basically agreed with measures 5 (n=381, 92.25%), 7 (n=385, 93.22%), 11 (n=372, 90.07%), 14 (n=372, 90.08%), 15 (n=386, 93.46%), 18 (n=379, 91.77%), 31 (n=375, 90.80%), and 35 (n=388, 93.95%). Even for measures 3, 4, 17, 23, and 28, 312 (75.54%), 322 (77.97%), 320 (77.48%), 298 (72.15%), and 286 (69.25%) of participants totally and basically agreed, respectively. Specifically, the rates of students totally agreeing with the 36 measures ranged from 76 (18.40%) to 147 (35.59%) and those basically agreeing with these measures varied between 210 (50.85%) and 286 (69.25%). This means that most of the participating students showed a positive attitude toward their experience of the use of chatbots.

**Table 3:** Descriptive analysis of user experience and satisfaction. Items 1-36 represent the 36 measures in the questionnaire (N=413 for each item).

Item		Minimum score	Maximum score	Mean score (SD)	Median score
**Conditional domain (mean 1.995, SD 0.718)**					
	1	1.000	4.000	1.908 (0.666)	2.000
	2	1.000	4.000	1.971 (0.686)	2.000
	3	1.000	4.000	2.048 (0.777)	2.000
	4	1.000	4.000	2.051 (0.731)	2.000
**Epistemic domain (mean 1.834, SD 0.654)**					
	5	1.000	4.000	1.738 (0.646)	2.000
	6	1.000	4.000	2.000 (0.690)	2.000
	7	1.000	4.000	1.765 (0.627)	2.000
**Functional domain (mean 1.762, SD 0.630)**					
	8	1.000	4.000	1.978 (0.648)	2.000
	9	1.000	4.000	1.891 (0.639)	2.000
	10	1.000	4.000	1.881 (0.606)	2.000
	11	1.000	4.000	1.881 (0.602)	2.000
	12	1.000	4.000	1.942 (0.647)	2.000
	13	1.000	4.000	1.927 (0.627)	2.000
	14	1.000	4.000	1.862 (0.629)	2.000
	15	1.000	4.000	1.794 (0.602)	2.000
	16	1.000	4.000	1.932 (0.631)	2.000
	17	1.000	4.000	2.046 (0.700)	2.000
	18	1.000	4.000	1.872 (0.596)	2.000
	19	1.000	4.000	1.896 (0.620)	2.000
	20	1.000	4.000	1.891 (0.639)	2.000
**Social domain (mean 1.998, SD 0.696)**					
	21	1.000	4.000	1.915 (0.639)	2.000
	22	1.000	4.000	1.998 (0.695)	2.000
	23	1.000	4.000	2.133 (0.775)	2.000
	24	1.000	4.000	1.944 (0.675)	2.000
**Emotional domain (mean 1.993, SD 0.683)**					
	25	1.000	4.000	1.976 (0.679)	2.000
	26	1.000	4.000	2.010 (0.686)	2.000
**Experience domain (mean 1.978, SD 0.639)**					
	27	1.000	4.000	1.913 (0.617)	2.000
	28	1.000	4.000	2.155 (0.751)	2.000
	29	1.000	4.000	2.019 (0.653)	2.000
	30	1.000	4.000	1.913 (0.593)	2.000
	31	1.000	4.000	1.891 (0.583)	2.000
**Satisfaction domain (mean 1.894, SD 0.617)**					
	32	1.000	4.000	1.901 (0.593)	2.000
	33	1.000	4.000	1.939 (0.634)	2.000
	34	1.000	4.000	1.947 (0.648)	2.000
	35	1.000	4.000	1.792 (0.595)	2.000
	36	1.000	4.000	1.889 (0.617)	2.000

### Correlation Between the 5 Domains and User Experience and Satisfaction

Table S4 in Multimedia Appendix 2 also demonstrates that the 5 domains are intimately correlated with user experience and satisfaction, that is, the former considerably contributes to the latter. Pearson correlation was used to determine the correlation between each of the 26 measures (1-26) in the 5 domains and each of the 10 measures (27-36) in overall user experience and satisfaction. Statistics showed that each of the former 26 measures is positively correlated with each of the latter 10 measures, with *P*<.001 for each correlation and all correlation coefficients varying from 0.459 to 0.844. This indicates that the functional, epistemic, emotional, social, and conditional values of health care chatbots contribute positively to overall user experience and satisfaction, as far as the 413 informants of this study are concerned.

### Differences in User Experience and Satisfaction by Gender and Grade

Table 4 illustrates the mean scores of all the 36 measures rated by males and females. The t test revealed that there was a significant difference between male ratings and female ratings, with the former being significantly higher than the latter (*P*<.001), as shown in Table 5. This implies that female participants were more positive in their experience of and satisfaction with health care chatbots compared to their male counterparts.

According to the *t* test (Table 6), there was a significant difference between freshmen’s ratings and sophomores’ ratings (*P*<.001), between freshmen’s ratings and first-year graduate candidates’ ratings (*P*<.001), between freshmen’s ratings and second-year graduate candidates’ ratings (*P*<.001), between sophomores’ ratings and juniors’ ratings (*P*=.004), between sophomores’ ratings and seniors’ ratings (*P*<.001), between sophomores’ ratings and second-year graduate candidates’ ratings (*P*<.001), between juniors’ ratings and seniors’ ratings (*P*<.001), between juniors’ ratings and first-year graduate candidates’ ratings (*P*=.01), between juniors’ ratings and second-year graduate candidates’ ratings (*P*<.001), between seniors’ ratings and first-year graduate candidates’ ratings (*P*<.001), between seniors’ ratings and second-year graduate candidates’ ratings (*P*<.001), and between first- and second-year graduate candidates’ ratings (*P*=.002). This indicates that freshmen had a better experience and greater satisfaction than sophomores, first-year graduate candidates, and second-year graduate candidates when communicating with health care chatbots for COVID-19–related information. Sophomores had a better experience and greater satisfaction than second-year graduate candidates but a less positive experience and lesser satisfaction than juniors and seniors. Juniors felt more positive than first- and second-year graduate candidates but less positive than seniors in their experience and satisfaction. Seniors had a better experience and greater satisfaction than first- and second-year graduate candidates. First-year graduate candidates felt more positive than second-year graduate candidates when engaged in conversations with the health care chatbots.

Overall, seniors were the most positive when expressing their experience of and satisfaction with health care chatbots, closely followed by freshmen and juniors. Slightly less positive, sophomores and first-year graduate candidates had similar experience and satisfaction. Second-year graduate candidates did not feel so positive as the other 5 grade categories.

**Table 4:** Mean scores of the 36 measures by gender.

Item	Mean score (SD) by males	Mean score (SD) by females
1	1.945 (0.803)	1.903 (0.643)
2	2.012 (0.782)	1.967 (0.671)
3	2.091 (0.867)	2.044 (0.764)
4	2.018 (0.805)	2.058 (0.720)
5	1.818 (0.772)	1.731 (0,624)
6	2.018 (0.828)	2.000 (0.667)
7	1.927 (0.813)	1.744 (0.591)
8	2.036 (0.769)	1.972 (0.628)
9	2.055 (0.826)	1.869 (0.602)
10	2.000 (0.816)	1.867 (0.566)
11	2.000 (0.754)	1.867 (0.576)
12	2.036 (0.838)	1.931 (0.613)
13	2.036 (0.838)	1.914 (0.587)
14	1.945 (0.780)	1.853 (0.603)
15	1.964 (0.860)	1.772 (0.549)
16	2.055 (0.848)	1.917 (0.590)
17	2.127 (0.862)	2.036 (0.673)
18	2.000 (0.793)	1.856 (0.558)
19	1.982 (0.871)	1.886 (0.573)
20	1.964 (0.816)	1.883 (0.608)
21	2.018 (0.871)	1.903 (0.595)
22	2.036 (0.881)	1.994 (0.663)
23	2.255 (0.927)	2.117 (0.749)
24	2.036 (0.793)	1.933 (0.655)
25	2.055 (0.826)	1.967 (0.654)
26	2.073 (0.836)	2.003 (0.661)
27	1.964 (0.769)	1.908 (0.591)
28	2.164 (0.918)	2.156 (0.723)
29	2.036 (0.793)	2.019 (0.630)
30	1.945 (0.756)	1.911 (0.565)
31	2.036 (0.769)	1.872 (0.547)
32	1.964 (0.816)	1.894 (0.552)
33	1.927 (0.790)	1.944 (0.607)
34	1.982 (0.805)	1.944 (0.621)
35	1.873 (0.795)	1.783 (0.559)
36	1.982 (0.805)	1.878 (0.583)

**Table 5:** Results of the *t* test of mean scores of the 36 measures by gender (*t* test *P*<.001).

Classification	Participants, n (%)	Minimum score	Maximum score	Mean score (SD)
Male	55 (13.32)	1.000	4.000	1.999 (0.051)
Female	358 (86.68)	1.000	4.000	1.931 (0.098)

**Table 6:** Results of the *t* test of mean scores of the 36 measures by grade.

Classification	Participants, n (%)	Minimum score	Maximum score	Mean score (SD)	Freshman *P* value	Sophomore *P* value	Junior *P* value	Senior *P* value	First-year graduate candidate *P* value	Second-year graduate candidate *P* value
Freshman	66	1.000	4.000	1.883 (0.114)	N/A^a^	<.001	.24	.08	<.001	<.001
Sophomore	72	1.000	4.000	1.989 (0.092)	<.001	N/A	.004	<.001	.81	<.001
Junior	110	1.000	4.000	1.925 (0.087)	.24	.004	N/A	.001	.01	<.001
Senior	68	1.000	4.000	1.853 (0.108)	.08	<.001	.001	N/A	<.001	<.001
First-year graduate candidate	52	1.000	4.000	1.992 (0.116)	<.001	.81	.001	<.001	N/A	.002
Second-year graduate candidate	45	1.000	4.000	2.069 (0.133)	<.001	<.001	<.001	<.001	.002	N/A

^a^N/A: not applicable.

## Discussion

### Principal Findings

Young people aged 17-35 years constitute a population that is considered particularly receptive to health care chatbots during the omicron waves of COVID-19 for self-diagnosis and information about the latest virus variants. The findings of this study bring into focus the effect of the functional, epistemic, emotional, social, and conditional values of health care chatbots on the user experience and satisfaction of this specific population. Our findings suggest a considerable positive impact of these values on their overall user experience and satisfaction and a positive correlation between these values and user experience and satisfaction. By conducting an online questionnaire survey in the midst of the repeated outbreaks of the COVID-19 pandemic, we found that all the participants basically had a positive experience and were thus satisfied with the selected health care chatbots due to their generally satisfactory services. Results of the statistics also showed different degrees of experience of and satisfaction with the chosen health care chatbots among the 5 grade categories of participants: Seniors were the most receptive to health care chatbots for COVID-19 self-diagnoses and information, while second-year graduate candidates were the least receptive; freshmen and juniors felt slightly more positive than sophomores and first-year graduate candidates when engaged in conversations with the chatbots. In addition, female informants showed a relatively more receptive attitude toward the selected chatbots than male respondents. One possible reason for the relatively low reception among second-year graduate candidates is that they basically belonged to the oldest age group and were comparatively less willing to accept the novel way of obtaining information through communicating with chatbots. Although there are no studies devoted to age-related differences in user experience and satisfaction, this aspect deserves further investigation.

In addition to the chatbots’ advantages, such as accessibility, cost-effectiveness, and flexibility [51], the functional, epistemic, emotional, social, and conditional values contributed to the overall pleasant experience and general satisfaction among the 413 respondents. According to statistics, the functional and epistemic values of the selected chatbots were the most important contributors to the students’ positive experience and overall satisfaction. Functional values are concerned with functional and utilitarian performance [52]. In this study, the informants believed that the chatbots could be aware of the consulting context to use suitable language to provide personalized services based on their specific needs [53]. Personalization is a crucial function of artificial intelligence–based applications [54]. The selected chatbots of this study provided the survey participants with such personalized services as feedback, health reports, alerts, and recommendations [22], dealing with diverse mental health issues bothering different people during the repeated resurgences of COVID-19 [46] and leading to a higher level of user experience and satisfaction [22,55]. In addition, we found that other functional values, including user-friendliness, ease of use, and performance (eg, timely, precise, accurate, and effective answering, error-handling capacity) [47], also contributed to the participants’ generally positive experience and overall satisfaction. Communicating with the health care chatbots offered student informants novelty and satisfied their desire for knowledge [49], too. The novel way of learning self-diagnoses and general information concerning COVID-19 and the latest virus variants led to a basically positive experience of and overall satisfaction with the health care chatbots on the part of the respondents. This is in tune with some extant studies [49,52,56].

The conditional, emotional, and social values played similar roles in providing the informants with good experience and general satisfaction. Facing numerous mental disorders caused by COVID-19 worldwide, people have suffered from stress, anxiety, depression, and other psychological problems [57]. As such, chatbots have been launched to psychologically assist people in many countries during COVID-19 [58]. Such particular conditions and situations of time, place, technology, and people’s mental state [59,60] promote the decision [61] made by the informants to resort to health care chatbots for self-diagnosis and the general information about COVID-19 and the latest virus variants. The survey participants found that the health care chatbots were available almost anytime and anyplace, providing faster health care services and reducing contact-induced risks. Thus, informed by Lee et al [62], we concluded that the conditional values of chatbots perceived by the participants in the face of the worldwide health emergency of COVID-19 positively influenced the user experience of and satisfaction with the health care chatbots. This finding is in line with recent studies [48,52].

As an emotional value of chatbots [48], enjoyment is an important element of chatbots [40]. The respondents of this study considered that communicating with the chatbots gave them an enjoyable feeling and considerably relieved them of stress, depression, and anxiety, as proven in recent studies [62,63]. The impact of enjoyment and delight on the user experience of chatbots [64], user adoption [65], and user satisfaction [19,66] has been proven by some studies. This feeling helped relieved the stress, depression, and anxiety [66] of the informants of this study during the critical period of repeated outbreaks of COVID-19, contributing to their positive experience of and overall satisfaction with the health care chatbots chosen for this research.

User experience during the human-product interaction results from all respects of user feelings (functional, emotional, social, etc) [67], each of which brings about a particular evaluation of the product or service concerned [68]. In this study, the social values of the health care chatbots were also perceived by the participants. They believed that the selected chatbots could fully engage them when they communicated with the chatbots for self-diagnosis and acquisition of general information concerning COVID-19 and the latest virus variants, thus satisfying their needs for communication, affection, and social belonging [69]. They thought that they felt the chatbots’ empathetic tones when conversing about COVID-19–related health care information and that their personal information would not be misused unreasonably and illegally. Such humanlike empathy and privacy protection led to a more positive outlook, a feeling of emotional backup, and a sense of social belonging on the part of the informants, establishing trust and emotional connection between them and the chatbots [69].

### Implications

Informed by Deneche and Warren’s [47] evaluation framework, Zhu et al’s [48] measures of variables, and TCV [49,70], this study established a new assessment framework to investigate the informants’ user experience of and satisfaction with the selected health care chatbots. It advanced the theory regarding the user experience of and satisfaction with health chatbots from the perspective of TCV, enriching previous studies that focus little on this aspect [48]. Although previous studies have examined the user experience of and satisfaction with health chatbots in terms of effectiveness, usability, and acceptability, personalization, enjoyment, and novelty, they have explored this topic drawing on TAT [19-23,40,41,63], for example, the Technology Acceptance Model (TAM) and the Unified Theory of Acceptance and Use of Technology Model (UTAUT). TAM and UTAUT are primarily concerned with the relationship between the user behavior and the quality and function of technology-empowered products, so these theories fail to provide a full account of the utilization of health care chatbots in various human-machine interaction settings, particularly in the context of the COVID-19–induced social distancing and even lockdown [48]. Comparing TAT with TCV, we found that the latter has a greater power of explanation: TCV comprehensively integrates a variety of value-oriented factors (functional emotional, epistemic, social, and conditional) into the account of the behaviors of users when engaging in communication with chatbots. Therefore, the user experience and satisfaction assessment model we established based on TCV is most likely to gain a better understanding of the user experience of and satisfaction with health care chatbots during the public health emergency of COVID-19 and other public health crises and natural disasters. In addition, the assessment scale of 36 items and 5 dimensions we newly developed is more comprehensive than Deneche and Warren’s [47] international assessment framework and Zhu et al’s [48] measures of variables, thereby having high reliability (Cronbach *α*=.986) and validity (KMO=0.980). Although many countries have provided chatbots to psychologically assist the public during the COVID-19–induced health emergency [58], almost no research has been conducted to study the user experience of and satisfaction with mental health chatbots during this pandemic [54]. This paper fills the gap in the extant literature.

On the practical facet, the new assessment framework of this research and the related findings can inspire artificial intelligence (AI) companies or scientific institutions to better design health care chatbots by giving top priority to the functional and epistemic values of these CAs while not neglecting their emotional, social, and conditional values. Health care chatbots integrating these 5 domains of values can enhance user experience and satisfaction. This paper also provides the governments of all countries with certain guidelines to choose and popularize health care chatbots in times of public health emergencies, such as COVID-19. As the first generation living with AI, we have the responsibility to design chatbots and make them ubiquitous and helpful to the whole society [69].

### Limitations

Several limitations may influence the generalization of the findings reported in this paper. Most importantly, some of our findings may be biased due to the selection of respondents. The higher percentage of female respondents may be related to this bias. Particularly, the slightly higher level of user experience of and satisfaction with the selected health care chatbots may be attributed to the slightly higher percentage of female respondents. Additionally, we did not ask whether respondents had previous experience of health care chatbots, so we were unable to clarify whether our findings were biased by a mixture of respondents both with and without prior experience in this aspect. Finally, the survey is cross-sectional and lacks comparison to a period unaffected by the COVID-19 pandemic or to a different time of the year, and the data were collected merely from 1 university. We were unable to ascertain that the findings of this study can be generalized to the same age group in other regions or countries. The generalizability and validity of the findings and the assessment framework of this study need to be examined in further studies.

### Conclusion

Government agencies worldwide have been providing the public with chatbots to psychologically assist them [58] in coping with a plethora of mental disorders caused by COVID-19 [57]. However, there is little focus on the user experience of and satisfaction with health care chatbots among young people in the literature. This study deals with the use of health care chatbots among young people (aged 17-35 years) in China, mainly investigating their user experience and satisfaction through a newly designed assessment framework. The findings illustrate that the functional, epistemic, emotional, social, and conditional domains in the new assessment framework all have a positive impact on the participants’ user experience and satisfaction. This paper advances the theory regarding the usability of health care chatbots, and chatbots for other purposes, enriching the literature. It also provides practical implications for chatbot designers and developers as well as for governments of all countries, especially in the critical period of the omicron waves of COVID-19 and other future public health crises.

## Appendix

Multimedia Appendix 1 Survey questionnaire.

Multimedia Appendix 2 Supplementary tables.

## Abbreviations

AI: artificial intelligence
CA: conversational agents
CET: College English Test
IS: information systems
KMO: Kaiser-Meyer-Olkin
TAM: Technology Acceptance Model
TAT: technology acceptance theories
TCV: theory of consumption values
TEM: Test for English Majors
UTAUT: Unified Theory of Acceptance and Use of Technology Model
