# Supplementary material for: Use of Health Care Chatbots Among Young People in China During the Omicron Wave of COVID-19: Evaluation of the User Experience of and Satisfaction With the Technology
Source: JMIR Hum Factors. 2022 Jun 9;9(2):e36831. doi: 10.2196/36831 (PMC9186498; doi:10.2196/36831)
Supplement: Multimedia Appendix 1 [file humanfactors_v9i2e36831_app1.docx]

### The Questionnaire Includes 36 Measures

A survey on the user experience of and satisfaction with health care chatbots among young people (17-35 years old) in China in the context of COVID-19.

Hello! I am a teacher at the School of Foreign Studies, Nantong University, and I am conducting a study on health care chatbots. I would like to get some information about chatbots on online medical platforms!

In the context of the ever-changing COVID-19 pandemic, you must be very concerned about the health of yourself and your family. Don’t worry! Health care chatbots can help you understand the dynamics of the epidemic and provide you with various information about personal protection.

To ensure the validity and reliability of the questionnaire, be sure to read the following instructions carefully before answering the questions:

1. When experiencing the use of health care chatbots, you need to ask various questions about COVID-19 (such as virus mutation, vaccination, personal travel, daily protection, etc.).

2. Please fill in the “Personal Information” part of the questionnaire truthfully.

3. In the “Health Literacy” section, do not consult the dictionary when meeting any new words.

4. Please finish the “Experience” part according to your own experience and satisfaction.

5. Please do finish all the questions before submitting the questionnaire.

Part I Personal Information

See Table 2

Part II Health Literacy


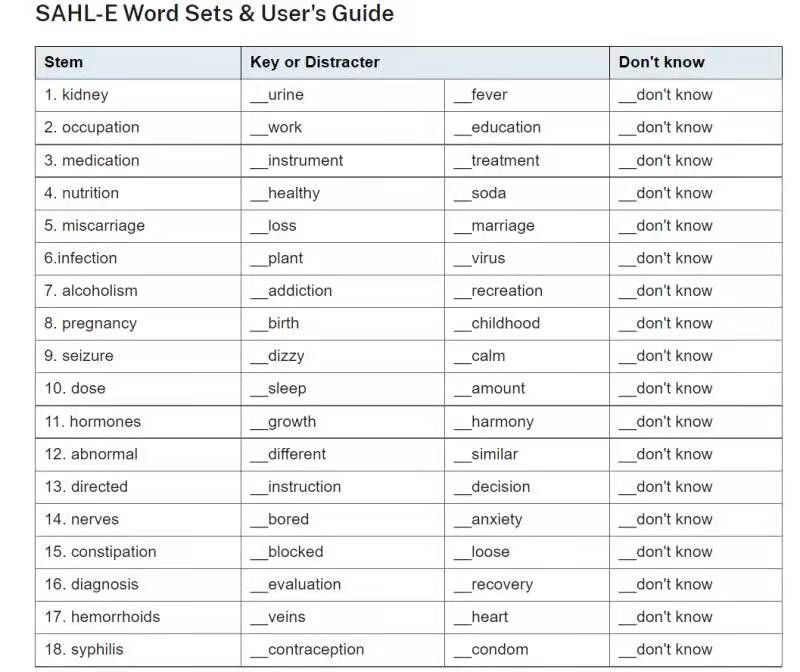


Part III User Experience and Satisfaction

1. In the context of the ever-changing epidemic, I try to obtain health care information related to the COVID-19 by communicating with chatbots at any time.

**￮** Totally agree **￮** Basically agree **￮** Totally disagree **￮** Basically disagree

1. In the context of the ever-changing epidemic, whether at home, at school, or elsewhere, I try to communicate with chatbots to obtain health care information related to the COVID-19.

**￮** Totally agree **￮** Basically agree **￮** Totally disagree **￮** Basically disagree

1. Under the current conditions of science and technology (network, computer, mobile phone, application, etc.), I think that it is completely possible to obtain health care information related to the COVID-19 by communicating with chatbots.

**￮** Totally agree **￮** Basically agree **￮** Totally disagree **￮** Basically disagree

1. The pandemic has affected my psychology to a certain extent, and I think that it is necessary to communicate with chatbots to obtain health care information related to the COVID-19 virus.

**￮** Totally agree **￮** Basically agree **￮** Totally disagree **￮** Basically disagree

1. I find communicating with chatbots very novel and attractive to get COVID-19 related health care information.

**￮** Totally agree **￮** Basically agree **￮** Totally disagree **￮** Basically disagree

1. I find that communicating with chatbots can meet my needs for access to health care information related to COVID-19.

**￮** Totally agree **￮** Basically agree **￮** Totally disagree **￮** Basically disagree

1. I find that communicating with chatbots can increase knowledge about health care related to COVID-19.

**￮** Totally agree **￮** Basically agree **￮** Totally disagree **￮** Basically disagree

1. I believe that the chatbot can accurately understand the questions I ask and the information I provide when communicating with the chatbot to gain health care knowledge related to COVID-19.

**￮** Totally agree **￮** Basically agree **￮** Totally disagree **￮** Basically disagree

1. I think chatbots are able to communicate using appropriately difficult, highly interactive language when I communicate with chatbots to acquire health care knowledge related to the coronavirus.

**￮** Totally agree **￮** Basically agree **￮** Totally disagree **￮** Basically disagree

1. I believe that the language used by the chatbot is appropriate for my age group when I communicate with the chatbot to gain health care knowledge related to COVID-19.

**￮** Totally agree **￮** Basically agree **￮** Totally disagree **￮** Basically disagree

1. I think chatbots are able to communicate fluently when I communicate with chatbots to gain health care knowledge related to COVID-19.

**￮** Totally agree **￮** Basically agree **￮** Totally disagree **￮** Basically disagree

1. I think chatbots are able to communicate in plain language when I communicate with chatbots to gain health care knowledge related to COVID-19.

**￮** Totally agree **￮** Basically agree **￮** Totally disagree **￮** Basically disagree

1. I think that when I ask the chatbot about COVID-19-related health care information, the chatbot is able to provide me with targeted answers based on the specific questions I ask, meeting my personal needs.

**￮** Totally agree **￮** Basically agree **￮** Totally disagree **￮** Basically disagree

1. When asking chatbots for COVID-19-related health care information, I find chatbots simple and easy to use.

**￮** Totally agree **￮** Basically agree **￮** Totally disagree **￮** Basically disagree

1. When asking chatbots for COVID-19-related health care information, I find that chatbots can respond in a timely manner.

**￮** Totally agree **￮** Basically agree **￮** Totally disagree **￮** Basically disagree

1. When asking chatbots for COVID-19-related health care information, I find that chatbots can answer accurately.

**￮** Totally agree **￮** Basically agree **￮** Totally disagree **￮** Basically disagree

1. When asking chatbots for COVID-19-related health care information, I find that chatbots can provide all the information I need.

**￮** Totally agree **￮** Basically agree **￮** Totally disagree **￮** Basically disagree

1. When asking chatbots for COVID-19-related health care information, I find that chatbots can provide reliable information.

**￮** Totally agree **￮** Basically agree **￮** Totally disagree **￮** Basically disagree

1. When asking chatbots for COVID-19-related health care information, I think chatbots can effectively deal with problems in communication, such as incomplete information input by users, incorrect text input, etc.

**￮** Totally agree **￮** Basically agree **￮** Totally disagree **￮** Basically disagree

1. When asking chatbots for COVID-19-related health care information, I find chatbots very efficient.

**￮** Totally agree **￮** Basically agree **￮** Totally disagree **￮** Basically disagree

1. When asking chatbots about COVID-19-related health care and mental health counseling, I am able to interact effectively and communicate fully with the bots.
   **￮** Totally agree **￮** Basically agree **￮** Totally disagree **￮** Basically disagree
2. When I ask the chatbot about health care and mental health counseling information about the COVID-19, the chatbot is able to use a tone of concern, which makes me spiritually and psychologically comforted.

**￮** Totally agree **￮** Basically agree **￮** Totally disagree **￮** Basically disagree

1. When I ask the chatbot about health care and mental health counseling information about the COVID-19, it was as if I was having a face-to-face conversation with a doctor.

**￮** Totally agree **￮** Basically agree **￮** Totally disagree **￮** Basically disagree

1. In my view, the information about health care and mental health counseling related to the COVID-19 that I communicate with the chatbot is only for non-commercial purposes such as medical diagnoses and scientific research, and will not be unreasonably or illegally used by online medical platforms (such as selling information).

**￮** Totally agree **￮** Basically agree **￮** Totally disagree **￮** Basically disagree

1. I think communicating with chatbots about health care, mental health counseling, etc. makes me relaxed and happy.

**￮** Totally agree **￮** Basically agree **￮** Totally disagree **￮** Basically disagree

1. I think that I can relieve psychological stress, anxiety, depression, and other emotions after communicating with chatbots about COVID-19-related health care, mental health counseling, etc.

**￮** Totally agree **￮** Basically agree **￮** Totally disagree **￮** Basically disagree

1. I think that the consultation services provided by chatbots on health care and mental health counseling related to the COVID-19 are professional and trustworthy.

**￮** Totally agree **￮** Basically agree **￮** Totally disagree **￮** Basically disagree

1. When communicating with chatbots for health care consultation and mental health counseling related to the COVID-19, I think the bot is empathetic.
    **￮** Totally agree **￮** Basically agree **￮** Totally disagree **￮** Basically disagree
2. After communicating with the chatbot for health care consultation and mental health counseling related to the COVID-19, I feel much better.

**￮** Totally agree **￮** Basically agree **￮** Totally disagree **￮** Basically disagree

1. After communicating with the chatbot for health care consultation and mental health counseling related to the COVID-19, I think that the overall use experience is very good.

**￮** Totally agree **￮** Basically agree **￮** Totally disagree **￮** Basically disagree

1. During communicating with the chatbot for health care consultation and mental health counseling related to the COVID-19, I am not disappointed with the service provided by the bot.

**￮** Totally agree **￮** Basically agree **￮** Totally disagree **￮** Basically disagree

1. I am very satisfied with the counseling services provided by the chatbot on health care, mental health counseling, etc. related to the COVID-19.

**￮** Totally agree **￮** Basically agree **￮** Totally disagree **￮** Basically disagree

1. I think using chatbots for COVID-19-related health care, mental health counseling, etc. is a very sensible choice.

**￮** Totally agree **￮** Basically agree **￮** Totally disagree **￮** Basically disagree

1. I will continue to use chatbots for COVID-19-related health care, mental health counseling, etc.

**￮** Totally agree **￮** Basically agree **￮** Totally disagree **￮** Basically disagree

1. I think that the performance of online chatbots in providing consultation services such as health care and mental health counseling will continuously be improved, so as to provide better health consultation services.

**￮** Totally agree **￮** Basically agree **￮** Totally disagree **￮** Basically disagree

1. I will recommend the chatbot to family members, classmates, friends, etc., to help them get timely consultation services related to the COVID-19 in terms of health care and mental health counseling.

**￮** Totally agree **￮** Basically agree **￮** Totally disagree **￮** Basically disagree
