# Supplementary material for: Use of Health Care Chatbots Among Young People in China During the Omicron Wave of COVID-19: Evaluation of the User Experience of and Satisfaction With the Technology
Source: JMIR Hum Factors. 2022 Jun 9;9(2):e36831. doi: 10.2196/36831 (PMC9186498; doi:10.2196/36831)
Supplement: Multimedia Appendix 2 [file humanfactors_v9i2e36831_app2.docx]

Table S1. Reliability of Data on User Experience and Satisfaction.

| **Reliability Statistics (Cronbach Alpha)** | | | |
| --- | --- | --- | --- |
| **Items** | **Corrected Item-Total Correlation (CITC)** | **Cronbach Alpha if Item Deleted** | **Cronbach α** |
| 1 | 0.729 | 0.986 | 0.986 |
| 2 | 0.748 | 0.986 |  |
| 3 | 0.718 | 0.986 |  |
| 4 | 0.667 | 0.987 |  |
| 5 | 0.721 | 0.986 |  |
| 6 | 0.761 | 0.986 |  |
| 7 | 0.791 | 0.986 |  |
| 8 | 0.839 | 0.986 |  |
| 9 | 0.843 | 0.986 |  |
| 10 | 0.841 | 0.986 |  |
| 11 | 0.840 | 0.986 |  |
| 12 | 0.823 | 0.986 |  |
| 13 | 0.862 | 0.986 |  |
| 14 | 0.830 | 0.986 |  |
| 15 | 0.787 | 0.986 |  |
| 16 | 0.845 | 0.986 |  |
| 17 | 0.836 | 0.986 |  |
| 18 | 0.869 | 0.986 |  |
| 19 | 0.807 | 0.986 |  |
| 20 | 0.833 | 0.986 |  |
| 21 | 0.871 | 0.986 |  |
| 22 | 0.821 | 0.986 |  |
| 23 | 0.804 | 0.986 |  |
| 24 | 0.775 | 0.986 |  |
| 25 | 0.853 | 0.986 |  |
| 26 | 0.849 | 0.986 |  |
| 27 | 0.861 | 0.986 |  |
| 28 | 0.784 | 0.986 |  |
| 29 | 0.850 | 0.986 |  |
| 30 | 0.873 | 0.986 |  |
| 31 | 0.844 | 0.986 |  |
| 32 | 0.848 | 0.986 |  |
| 33 | 0.855 | 0.986 |  |
| 34 | 0.849 | 0.986 |  |
| 35 | 0.810 | 0.986 |  |
| 36 | 0.869 | 0.986 |  |
| Cronbach α (Standardized): 0.987 | | | |

Table S2. Validity of Data on User Experience and Satisfaction.

| **Validity Analysis** | | | | |
| --- | --- | --- | --- | --- |
| **Items** | **Factor Loadings** | | | **Communalities** |
|  | **Factor 1** | **Factor 2** | **Factor 3** |  |
| 1 | 0.406 | 0.267 | 0.687 | 0.707 |
| 2 | 0.381 | 0.309 | 0.705 | 0.737 |
| 3 | 0.333 | 0.309 | 0.709 | 0.709 |
| 4 | 0.193 | 0.353 | 0.736 | 0.703 |
| 5 | 0.631 | 0.214 | 0.434 | 0.632 |
| 6 | 0.400 | 0.397 | 0.590 | 0.666 |
| 7 | 0.708 | 0.263 | 0.409 | 0.738 |
| 8 | 0.499 | 0.493 | 0.491 | 0.734 |
| 9 | 0.637 | 0.412 | 0.418 | 0.750 |
| 10 | 0.668 | 0.384 | 0.409 | 0.761 |
| 11 | 0.679 | 0.389 | 0.384 | 0.761 |
| 12 | 0.611 | 0.447 | 0.367 | 0.708 |
| 13 | 0.629 | 0.486 | 0.370 | 0.769 |
| 14 | 0.682 | 0.401 | 0.345 | 0.745 |
| 15 | 0.799 | 0.268 | 0.272 | 0.784 |
| 16 | 0.629 | 0.482 | 0.340 | 0.743 |
| 17 | 0.476 | 0.552 | 0.436 | 0.722 |
| 18 | 0.704 | 0.447 | 0.334 | 0.807 |
| 19 | 0.600 | 0.462 | 0.327 | 0.681 |
| 20 | 0.614 | 0.532 | 0.272 | 0.733 |
| 21 | 0.637 | 0.526 | 0.327 | 0.789 |
| 22 | 0.345 | 0.702 | 0.388 | 0.762 |
| 23 | 0.274 | 0.732 | 0.409 | 0.779 |
| 24 | 0.416 | 0.609 | 0.317 | 0.645 |
| 25 | 0.446 | 0.715 | 0.299 | 0.800 |
| 26 | 0.383 | 0.760 | 0.316 | 0.823 |
| 27 | 0.539 | 0.640 | 0.287 | 0.783 |
| 28 | 0.210 | 0.799 | 0.369 | 0.819 |
| 29 | 0.362 | 0.749 | 0.361 | 0.822 |
| 30 | 0.575 | 0.640 | 0.264 | 0.811 |
| 31 | 0.582 | 0.621 | 0.220 | 0.773 |
| 32 | 0.582 | 0.630 | 0.217 | 0.782 |
| 33 | 0.539 | 0.673 | 0.231 | 0.797 |
| 34 | 0.540 | 0.604 | 0.309 | 0.752 |
| 35 | 0.766 | 0.383 | 0.216 | 0.780 |
| 36 | 0.619 | 0.550 | 0.313 | 0.784 |
| Eigenvalues (Initial) | 24.768 | 1.222 | 1.103 | - |
| % of Variance (Initial) | 68.799% | 3.394% | 3.065% | - |
| % of Cum. Variance (Initial) | 68.799% | 72.192% | 75.257% | - |
| Eigenvalues (Rotated) | 10.954 | 10.108 | 6.031 | - |
| % of Variance (Rotated) | 30.428% | 28.077% | 16.752% | - |
| % of Cum. Variance (Rotated) | 30.428% | 58.505% | 75.257% | - |
| KMO | 0.980 | | | - |
| Bartlett's Test of Sphericity (Chi-Square) | 18279.068 | | | - |
| *df* | 630 | | | - |
| *P* value | <.001 | | | - |

Table S3. Frequency of Each Rating of the 36 measures of User Experience and Satisfaction. Items 1-6 represent the 36 measures in the questionnaire.

| **Frequency** | | | | |
| --- | --- | --- | --- | --- |
| **Items** | **Categories** | **N of Sample** | **Percent (%)** | **Cumulative Percent (%)** |
| 1 | totally agree | 101 | 24.46 | 24.46 |
|  | basically agree | 260 | 62.95 | 87.41 |
|  | basically disagree | 41 | 9.93 | 97.34 |
|  | totally disagree | 11 | 2.66 | 100.00 |
| 2 | totally agree | 95 | 23.00 | 23.00 |
|  | basically agree | 243 | 58.84 | 81.84 |
|  | basically disagree | 67 | 16.22 | 98.06 |
|  | totally disagree | 8 | 1.94 | 100.00 |
| 3 | totally agree | 98 | 23.73 | 23.73 |
|  | basically agree | 214 | 51.82 | 75.54 |
|  | basically disagree | 84 | 20.34 | 95.88 |
|  | totally disagree | 17 | 4.12 | 100.00 |
| 4 | totally agree | 85 | 20.58 | 20.58 |
|  | basically agree | 237 | 57.38 | 77.97 |
|  | basically disagree | 76 | 18.40 | 96.37 |
|  | totally disagree | 15 | 3.63 | 100.00 |
| 5 | totally agree | 147 | 35.59 | 35.59 |
|  | basically agree | 234 | 56.66 | 92.25 |
|  | basically disagree | 25 | 6.05 | 98.31 |
|  | totally disagree | 7 | 1.69 | 100.00 |
| 6 | totally agree | 90 | 21.79 | 21.79 |
|  | basically agree | 241 | 58.35 | 80.15 |
|  | basically disagree | 74 | 17.92 | 98.06 |
|  | totally disagree | 8 | 1.94 | 100.00 |
| 7 | totally agree | 133 | 32.20 | 32.20 |
|  | basically agree | 252 | 61.02 | 93.22 |
|  | basically disagree | 20 | 4.84 | 98.06 |
|  | totally disagree | 8 | 1.94 | 100.00 |
| 8 | totally agree | 84 | 20.34 | 20.34 |
|  | basically agree | 261 | 63.20 | 83.54 |
|  | basically disagree | 61 | 14.77 | 98.31 |
|  | totally disagree | 7 | 1.69 | 100.00 |
| 9 | totally agree | 103 | 24.94 | 24.94 |
|  | basically agree | 258 | 62.47 | 87.41 |
|  | basically disagree | 46 | 11.14 | 98.55 |
|  | totally disagree | 6 | 1.45 | 100.00 |
| 10 | totally agree | 97 | 23.49 | 23.49 |
|  | basically agree | 274 | 66.34 | 89.83 |
|  | basically disagree | 36 | 8.72 | 98.55 |
|  | totally disagree | 6 | 1.45 | 100.00 |
| 11 | totally agree | 96 | 23.24 | 23.24 |
|  | basically agree | 276 | 66.83 | 90.07 |
|  | basically disagree | 35 | 8.47 | 98.55 |
|  | totally disagree | 6 | 1.45 | 100.00 |
| 12 | totally agree | 92 | 22.28 | 22.28 |
|  | basically agree | 260 | 62.95 | 85.23 |
|  | basically disagree | 54 | 13.08 | 98.31 |
|  | totally disagree | 7 | 1.69 | 100.00 |
| 13 | totally agree | 91 | 22.03 | 22.03 |
|  | basically agree | 267 | 64.65 | 86.68 |
|  | basically disagree | 49 | 11.86 | 98.55 |
|  | totally disagree | 6 | 1.45 | 100.00 |
| 14 | totally agree | 106 | 25.67 | 25.67 |
|  | basically agree | 266 | 64.41 | 90.07 |
|  | basically disagree | 33 | 7.99 | 98.06 |
|  | totally disagree | 8 | 1.94 | 100.00 |
| 15 | totally agree | 119 | 28.81 | 28.81 |
|  | basically agree | 267 | 64.65 | 93.46 |
|  | basically disagree | 20 | 4.84 | 98.31 |
|  | totally disagree | 7 | 1.69 | 100.00 |
| 16 | totally agree | 90 | 21.79 | 21.79 |
|  | basically agree | 268 | 64.89 | 86.68 |
|  | basically disagree | 48 | 11.62 | 98.31 |
|  | totally disagree | 7 | 1.69 | 100.00 |
| 17 | totally agree | 83 | 20.10 | 20.10 |
|  | basically agree | 237 | 57.38 | 77.48 |
|  | basically disagree | 84 | 20.34 | 97.82 |
|  | totally disagree | 9 | 2.18 | 100.00 |
| 18 | totally agree | 95 | 23.00 | 23.00 |
|  | basically agree | 284 | 68.77 | 91.77 |
|  | basically disagree | 26 | 6.30 | 98.06 |
|  | totally disagree | 8 | 1.94 | 100.00 |
| 19 | totally agree | 96 | 23.24 | 23.24 |
|  | basically agree | 271 | 65.62 | 88.86 |
|  | basically disagree | 39 | 9.44 | 98.31 |
|  | totally disagree | 7 | 1.69 | 100.00 |
| 20 | totally agree | 100 | 24.21 | 24.21 |
|  | basically agree | 267 | 64.65 | 88.86 |
|  | basically disagree | 37 | 8.96 | 97.82 |
|  | totally disagree | 9 | 2.18 | 100.00 |
| 21 | totally agree | 94 | 22.76 | 22.76 |
|  | basically agree | 269 | 65.13 | 87.89 |
|  | basically disagree | 41 | 9.93 | 97.82 |
|  | totally disagree | 9 | 2.18 | 100.00 |
| 22 | totally agree | 90 | 21.79 | 21.79 |
|  | basically agree | 244 | 59.08 | 80.87 |
|  | basically disagree | 69 | 16.71 | 97.58 |
|  | totally disagree | 10 | 2.42 | 100.00 |
| 23 | totally agree | 80 | 19.37 | 19.37 |
|  | basically agree | 218 | 52.78 | 72.15 |
|  | basically disagree | 95 | 23.00 | 95.16 |
|  | totally disagree | 20 | 4.84 | 100.00 |
| 24 | totally agree | 97 | 23.49 | 23.49 |
|  | basically agree | 251 | 60.77 | 84.26 |
|  | basically disagree | 56 | 13.56 | 97.82 |
|  | totally disagree | 9 | 2.18 | 100.00 |
| 25 | totally agree | 91 | 22.03 | 22.03 |
|  | basically agree | 250 | 60.53 | 82.57 |
|  | basically disagree | 63 | 15.25 | 97.82 |
|  | totally disagree | 9 | 2.18 | 100.00 |
| 26 | totally agree | 86 | 20.82 | 20.82 |
|  | basically agree | 246 | 59.56 | 80.39 |
|  | basically disagree | 72 | 17.43 | 97.82 |
|  | totally disagree | 9 | 2.18 | 100.00 |
| 27 | totally agree | 92 | 22.28 | 22.28 |
|  | basically agree | 271 | 65.62 | 87.89 |
|  | basically disagree | 44 | 10.65 | 98.55 |
|  | totally disagree | 6 | 1.45 | 100.00 |
| 28 | totally agree | 76 | 18.40 | 18.40 |
|  | basically agree | 210 | 50.85 | 69.25 |
|  | basically disagree | 114 | 27.60 | 96.85 |
|  | totally disagree | 13 | 3.15 | 100.00 |
| 29 | totally agree | 76 | 18.40 | 18.40 |
|  | basically agree | 261 | 63.20 | 81.60 |
|  | basically disagree | 68 | 16.46 | 98.06 |
|  | totally disagree | 8 | 1.94 | 100.00 |
| 30 | totally agree | 86 | 20.82 | 20.82 |
|  | basically agree | 283 | 68.52 | 89.35 |
|  | basically disagree | 38 | 9.20 | 98.55 |
|  | totally disagree | 6 | 1.45 | 100.00 |
| 31 | totally agree | 89 | 21.55 | 21.55 |
|  | basically agree | 286 | 69.25 | 90.80 |
|  | basically disagree | 32 | 7.75 | 98.55 |
|  | totally disagree | 6 | 1.45 | 100.00 |
| 32 | totally agree | 89 | 21.55 | 21.55 |
|  | basically agree | 282 | 68.28 | 89.83 |
|  | basically disagree | 36 | 8.72 | 98.55 |
|  | totally disagree | 6 | 1.45 | 100.00 |
| 33 | totally agree | 88 | 21.31 | 21.31 |
|  | basically agree | 270 | 65.38 | 86.68 |
|  | basically disagree | 47 | 11.38 | 98.06 |
|  | totally disagree | 8 | 1.94 | 100.00 |
| 34 | totally agree | 90 | 21.79 | 21.79 |
|  | basically agree | 263 | 63.68 | 85.47 |
|  | basically disagree | 52 | 12.59 | 98.06 |
|  | totally disagree | 8 | 1.94 | 100.00 |
| 35 | totally agree | 118 | 28.57 | 28.57 |
|  | basically agree | 270 | 65.38 | 93.95 |
|  | basically disagree | 18 | 4.36 | 98.31 |
|  | totally disagree | 7 | 1.69 | 100.00 |
| 36 | totally agree | 97 | 23.49 | 23.49 |
|  | basically agree | 272 | 65.86 | 89.35 |
|  | basically disagree | 37 | 8.96 | 98.31 |
|  | totally disagree | 7 | 1.69 | 100.00 |
| Total | | 413 | 100.0 | 100.0 |

Table S4. Correlation Between Measures (1-26) in the Five domains and Measures (27-36) in User Experience and Satisfaction

| **Pearson Correlation** | | | | | | | | | | | | | | | | | | | | | | | | | | | |
| --- | --- | --- | --- | --- | --- | --- | --- | --- | --- | --- | --- | --- | --- | --- | --- | --- | --- | --- | --- | --- | --- | --- | --- | --- | --- | --- | --- |
|  |  | **1** | **2** | **3** | **4** | **5** | **6** | **7** | **8** | **9** | **10** | **11** | **12** | **13** | **14** | **15** | **16** | **17** | **18** | **19** | **20** | **21** | **22** | **23** | **24** | **25** | **26** |
| **27** | **Coefficient** | 0.583** | 0.614** | 0.611** | 0.516** | 0.631** | 0.690** | 0.693** | 0.718** | 0.690** | 0.719** | 0.698** | 0.692** | 0.756** | 0.694** | 0.696** | 0.739** | 0.700** | 0.769** | 0.680** | 0.746** | 0.757** | 0.724** | 0.714** | 0.682** | 0.783** | 0.764** |
|  | ***P* value** | <.001 | <.001 | <.001 | <.001 | <.001 | <.001 | <.001 | <.001 | <.001 | <.001 | <.001 | <.001 | <.001 | <.001 | <.001 | <.001 | <.001 | <.001 | <.001 | <.001 | <.001 | <.001 | <.001 | <.001 | <.001 | <.001 |
| **28** | **Coefficient** | 0.529** | 0.570** | 0.582** | 0.574** | 0.459** | 0.628** | 0.552** | 0.691** | 0.618** | 0.644** | 0.616** | 0.638** | 0.643** | 0.595** | 0.500** | 0.632** | 0.688** | 0.636** | 0.613** | 0.663** | 0.655** | 0.778** | 0.765** | 0.659** | 0.732** | 0.775** |
|  | ***P* value** | <.001 | <.001 | <.001 | <.001 | <.001 | <.001 | <.001 | <.001 | <.001 | <.001 | <.001 | <.001 | <.001 | <.001 | <.001 | <.001 | <.001 | <.001 | <.001 | <.001 | <.001 | <.001 | <.001 | <.001 | <.001 | <.001 |
| **29** | **Coefficient** | 0.596** | 0.603** | 0.610** | 0.603** | 0.605** | 0.679** | 0.639** | 0.724** | 0.697** | 0.674** | 0.648** | 0.703** | 0.703** | 0.674** | 0.590** | 0.680** | 0.698** | 0.680** | 0.634** | 0.686** | 0.719** | 0.775** | 0.728** | 0.679** | 0.817** | 0.844** |
|  | ***P* value** | <.001 | <.001 | <.001 | <.001 | <.001 | <.001 | <.001 | <.001 | <.001 | <.001 | <.001 | <.001 | <.001 | <.001 | <.001 | <.001 | <.001 | <.001 | <.001 | <.001 | <.001 | <.001 | <.001 | <.001 | <.001 | <.001 |
| **30** | **Coefficient** | 0.638** | 0.621** | 0.567** | 0.537** | 0.663** | 0.659** | 0.708** | 0.735** | 0.725** | 0.721** | 0.712** | 0.689** | 0.741** | 0.735** | 0.643** | 0.704** | 0.699** | 0.758** | 0.714** | 0.750** | 0.788** | 0.706** | 0.696** | 0.685** | 0.755** | 0.766** |
|  | ***P* value** | <.001 | <.001 | <.001 | <.001 | <.001 | <.001 | <.001 | <.001 | <.001 | <.001 | <.001 | <.001 | <.001 | <.001 | <.001 | <.001 | <.001 | <.001 | <.001 | <.001 | <.001 | <.001 | <.001 | <.001 | <.001 | <.001 |
| **3** | **Coefficient** | 0.599** | 0.599** | 0.536** | 0.526** | 0.588** | 0.610** | 0.653** | 0.675** | 0.717** | 0.719** | 0.724** | 0.697** | 0.709** | 0.687** | 0.675** | 0.705** | 0.708** | 0.749** | 0.673** | 0.724** | 0.770** | 0.700** | 0.703** | 0.688** | 0.748** | 0.718** |
|  | ***P* value** | <.001 | <.001 | <.001 | <.001 | <.001 | <.001 | <.001 | <.001 | <.001 | <.001 | <.001 | <.001 | <.001 | <.001 | <.001 | <.001 | <.001 | <.001 | <.001 | <.001 | <.001 | <.001 | <.001 | <.001 | <.001 | <.001 |
| **32** | **Coefficient** | 0.573** | 0.602** | 0.563** | 0.527** | 0.623** | 0.629** | 0.648** | 0.683** | 0.702** | 0.703** | 0.715** | 0.706** | 0.738** | 0.698** | 0.676** | 0.734** | 0.689** | 0.713** | 0.665** | 0.747** | 0.747** | 0.712** | 0.662** | 0.671** | 0.724** | 0.712** |
|  | ***P* value** | <.001 | <.001 | <.001 | <.001 | <.001 | <.001 | <.001 | <.001 | <.001 | <.001 | <.001 | <.001 | <.001 | <.001 | <.001 | <.001 | <.001 | <.001 | <.001 | <.001 | <.001 | <.001 | <.001 | <.001 | <.001 | <.001 |
| **33** | **Coefficient** | 0.602** | 0.594** | 0.568** | 0.520** | 0.655** | 0.622** | 0.648** | 0.695** | 0.709** | 0.696** | 0.694** | 0.672** | 0.692** | 0.728** | 0.635** | 0.712** | 0.701** | 0.738** | 0.669** | 0.751** | 0.719** | 0.722** | 0.708** | 0.724** | 0.753** | 0.766** |
|  | ***P* value** | <.001 | <.001 | <.001 | <.001 | <.001 | <.001 | <.001 | <.001 | <.001 | <.001 | <.001 | <.001 | <.001 | <.001 | <.001 | <.001 | <.001 | <.001 | <.001 | <.001 | <.001 | <.001 | <.001 | <.001 | <.001 | <.001 |
| **34** | **Coefficient** | 0.636** | 0.642** | 0.588** | 0.580** | 0.646** | 0.619** | 0.638** | 0.668** | 0.713** | 0.671** | 0.687** | 0.670** | 0.720** | 0.720** | 0.656** | 0.716** | 0.712** | 0.699** | 0.675** | 0.708** | 0.752** | 0.674** | 0.701** | 0.687** | 0.748** | 0.744** |
|  | ***P* value** | <.001 | <.001 | <.001 | <.001 | <.001 | <.001 | <.001 | <.001 | <.001 | <.001 | <.001 | <.001 | <.001 | <.001 | <.001 | <.001 | <.001 | <.001 | <.001 | <.001 | <.001 | <.001 | <.001 | <.001 | <.001 | <.001 |
| **35** | **Coefficient** | 0.601** | 0.592** | 0.536** | 0.482** | 0.704** | 0.591** | 0.752** | 0.624** | 0.687** | 0.719** | 0.710** | 0.668** | 0.694** | 0.726** | 0.746** | 0.686** | 0.617** | 0.773** | 0.657** | 0.687** | 0.694** | 0.603** | 0.539** | 0.635** | 0.702** | 0.664** |
|  | ***P* value** | <.001 | <.001 | <.001 | <.001 | <.001 | <.001 | <.001 | <.001 | <.001 | <.001 | <.001 | <.001 | <.001 | <.001 | <.001 | <.001 | <.001 | <.001 | <.001 | <.001 | <.001 | <.001 | <.001 | <.001 | <.001 | <.001 |
| **36** | **Coefficient** | 0.631** | 0.641** | 0.593** | 0.589** | 0.652** | 0.662** | 0.710** | 0.680** | 0.708** | 0.724** | 0.736** | 0.695** | 0.738** | 0.748** | 0.741** | 0.722** | 0.725** | 0.753** | 0.705** | 0.727** | 0.764** | 0.701** | 0.691** | 0.667** | 0.770** | 0.754** |
|  | ***P* value** | <.001 | <.001 | <.001 | <.001 | <.001 | <.001 | <.001 | <.001 | <.001 | <.001 | <.001 | <.001 | <.001 | <.001 | <.001 | <.001 | <.001 | <.001 | <.001 | <.001 | <.001 | <.001 | <.001 | <.001 | <.001 | <.001 |
| *** *p*<0.05 ** *p*<0.01** | | | | | | | | | | | | | | | | | | | | | | | | | | | |
